# Food protein induced enterocolitis syndrome caused by rice beverage

**DOI:** 10.1186/1824-7288-39-31

**Published:** 2013-05-14

**Authors:** Lucia Caminiti, Giuseppina Salzano, Giuseppe Crisafulli, Federica Porcaro, Giovanni Battista Pajno

**Affiliations:** 1 Department of Pediatrics, University of Messina, Messina, Italy

**Keywords:** Allergy, Enterocolitis, Rice beverage, Proteins

## Abstract

Food protein-induced enterocolitis syndrome (FPIES) is an uncommon and potentially severe non IgE-mediated gastrointestinal food allergy. It is usually caused by cow’s milk or soy proteins, but may also be triggered by ingestion of solid foods. The diagnosis is made on the basis of clinical history and symptoms. Management of acute phase requires fluid resuscitation and intravenous steroids administration, but avoidance of offending foods is the only effective therapeutic option.

Infant with FPIES presented to our emergency department with vomiting, watery stools, hypothension and metabolic acidosis after ingestion of rice beverage. Intravenous fluids and steroids were administered with good clinical response. Subsequently, a double blind placebo control food challenge (DBPCFC) was performed using rice beverage and hydrolyzed formula (eHF) as placebo. The “rice based formula” induced emesis, diarrhoea and lethargy. Laboratory investigations reveal an increase of absolute count of neutrophils and the presence of faecal eosinophils. The patient was treated with both intravenous hydration and steroids. According to Powell criteria, oral food challenge was considered positive and diagnosis of FPIES induced by rice beverage was made. Patient was discharged at home with the indication to avoid rice and any rice beverage as well as to reintroduce hydrolyzed formula. A case of FPIES induced by rice beverage has never been reported. The present case clearly shows that also beverage containing rice proteins can be responsible of FPIES. For this reason, the use of rice beverage as cow’s milk substitute for the treatment of non IgE-mediated food allergy should be avoided.

## Background

Food allergies are prevalent in the first 2 years of life and are usually IgE-mediated hypersensitivity reactions. Food protein-induced enterocolitis syndrome (FPIES) is a relatively rare and severe form of non IgE-mediated gastrointestinal food hypersensitivity reaction [1]. It is frequently misdiagnosed and unrecognized at the initial presentation. Typical form of FPIES is characterized by a clinical onset before nine-month of age with food-induced severe vomiting, bloody diarrhoea, abdominal distension, dehydration, lethargy, pallor and metabolic acidosis. Symptoms occur 1 – 5 hours after ingesting the offending food. While the most common provoking foods are cow’s milk and soy protein based formula, FPIES was also documented with other foods such as rice, fish, wheat and poultry. To some extent children with FPIES reacted to more than one food [2,3]. Diagnosis is based on history, clinical symptoms and exclusion of other causes, i.e. metabolic, toxicologic and intestinal etiologies.

Oral food challenge with 0.3 – 0.6 g/Kg of the suspected allergen can be used to establish diagnosis when history is unclear and to determine whether FPIES has resolved [4]. The majority of children will outgrow their FPIES by about three to four years of age. Avoidance of the offending food is currently the only specific treatment of FPIES.

Management of acute episode requires continued fluid resuscitation and intravenous steroids. Epinephrine is not recommended but it should be useful for potential hypotension and shock.

In this report, we describe the case of a Caucasian 8 month-old male who developed FPIES after administration of rice beverage.

## Case presentation

We report the case of a full term male patient who was initially breastfed. At 3 months a cow’s milk based formula was introduced to supplement breastfeeding because of reduction of mother’s milk production. At 4 months of age patient developed episodes of pallor, vomiting, diarrhoea and lethargy. Food protein induced enterocolitis syndrome caused by cow’s milk (CM) was diagnosed and an extensively bovine whey hydrolysed formula (eHF) was introduced; this formula was well tolerated and it was the only food used up to 7 months of age when he was admitted to our paediatric allergy unit for further evaluations. At admission the child’s physical examination was normal, weight was 1 SD below the median. Evaluation included urinalysis, stool culture, metabolic screening that resulted negative. The results of skin prick testing to CM proteins like β–lactoglobulin, α–lactalbumin and casein (Lofarma – Milan Italy) were negative as well as atopy patch testing. Total IgE was 8 KU/L, specific IgE to CM was slightly increased (0.85 KU/L) and there was no peripheral eosinophilia. We performed an oral food challenge with CM, which resulted positive: the increasing dose of 20 ml caused vomiting, diarrhoea and hematochezia; therefore eHF as CM substitute was reintroduced. One month later, the patient had repeated episodes of vomiting with pallor for four days, thereafter he referred again to our emergency department: he was hypothensive and acidotic because of vomiting and watery stools. He required 200 ml intravenous fluid infusion in 12 hours and intravenous steroids. The history showed that a rice based beverage was introduced by parents without any medical advice or consultation. The parents of patient believed rice like hypoallergenic food. After the child well recovered, we performed a double blind placebo controlled food challenge (DBPCFC). Preliminarily eHf was administered as placebo following the criteria of oral food challenge (OFC) for non IgE mediated FPIES allergy [5]; subsequently the challenge was carried out with rice beverage: until 48 mL whole rice beverage was given. We administered the dose gradually in 3 equal portions over a period of 45 minute. The “rice based formula” induced emesis, diarrhoea and lethargy. A complete blood count performed before challenge resulted in 844 mm^3^ peripheral neutrophil counts, compared with the one performed 6 hours after the challenge (neutrophils 5946 mm^3^) it indicated an increase of 5102 cells/mm^3^ in absolute neutrophils count after the challenge. Laboratory investigations detected also the presence of faecal neutrophils and eosinophils. The patient was treated with both intravenous hydration and steroids. According to the Powell criteria [6] the challenge was considered positive (Table 1). The patient recovered after 24 hours after the OFC and he was discharged at home with the diagnosis of protein-induced enterocolitis syndrome (FPIES) and the indication to avoid rice and any rice beverage. eHF was reintroduced and currently the patient is well. Subsequently he started successfully the weaning.

**Table 1:** **Criteria of positivity for oral food challenge according to Powell [**[6]**]**

**Preparatory steps**	Observe/test for criteria*
Emergency therapies in place (i.e. intravenous access)	Vomiting, diarrhoea
Verify normal weight gain and lack of symptoms while avoiding potential causal protein	Faecal blood (frank or occult)
Baseline and follow-up stool samples	Faecal leucocytes
Baseline peripheral blood PNM count	Faecal eosinophils
Repeat peripheral blood PNM count 6 h after ingestion	Increase of neutrophils >3.500/mm^3^

*Oral food challenge is considered positive when three or more criteria are satisfied.

## Conclusions

FPIES related to rice is a well recognized entity [7]. Pediatricians should be aware that rice beverage not only has the potential to cause FPIES, but also that such reactions tend to be severe as those caused by cow’s milk [8] or soy [9]. A case of FPIES induced by rice beverage has never been reported. The present case clearly shows that also beverage containing rice proteins can be responsible of FPIES. Foods that may perceived as hypoallergenic are thought no triggers of FPIES; on the other hand rice is a common cause of FPIES and the use of rice beverage as cow’s milk substitute for the treatment of non IgE-mediated food allergy should be avoided. Of note, both early diagnosis and correct management of disease would ensure that appropriate dietary advice is given to families, thereby reducing the risk of repeated reactions, inappropriate interventions, and frequent hospitalizations. Moreover, rice beverages are “formulas” with low content of proteins, therefore children feeding only this food are at risk of low protein intake [10].

Rice is considered a trigger of FPIES and a reaction to rice beverage should be considered as a possibility.

## Consent

Written informed consent was obtained from the parents of the patient for publication of this Case report. A copy of the written consent is available for review by the Editor-in-Chief of this journal.

## Abbreviations

FPIES: food protein induced enterocolitis; CM: cow’s milk; eHF: hydrolysed formula; DBPCFC: double blind placebo controlled food challenge.

## Competing interests

The Authors declare that they have no competing interests.

## Authors’ contribution

GBP, GS have been involved in drafting the manuscript. LC, FP, GC have been involved in collecting the clinical data and reviewing the scientific literature. GBP has revised critically conception and design of the manuscript. All the Authors read and approved the final manuscript.
